# An underwater observation dataset for fish classification and fishery assessment

**DOI:** 10.1038/sdata.2018.190

**Published:** 2018-10-09

**Authors:** Erin McCann, Liling Li, Kevin Pangle, Nicholas Johnson, Jesse Eickholt

**Affiliations:** 1 Department of Biology, Central Michigan University, Mt. Pleasant, MI 48859, USA; 2 Department of Computer Science, Central Michigan University, Mt. Pleasant, MI, 48859, USA; 3 Great Lakes Science Center, U.S. Geological Survey, Hammond Bay Biological Station, Millersburg, MI, 49759, USA

**Keywords:** Invasive species, Freshwater ecology

## Abstract

Using Dual-Frequency Identification Sonar (DIDSON), fishery acoustic observation data was collected from the Ocqueoc River, a tributary of Lake Huron in northern Michigan, USA. Data were collected March through July 2013 and 2016 and included the identification, via technology or expert analysis, of eight fish species as they passed through the DIDSON’s field of view. A set of short DIDSON clips containing identified fish was curated. Additionally, two other datasets were created that include visualizations of the acoustic data and longer DIDSON clips. These datasets could complement future research characterizing the abundance and behavior of valued fishes such as walleye (*Sander vitreus*) or white sucker (*Catostomus commersonii*) or invasive fishes such as sea lamprey (*Petromyzon marinus*) or European carp (*Cyprinus carpio*). Given the abundance of DIDSON data and the fact that a portion of it is labeled, these data could aid in the creation of machine learning tools from DIDSON data, particularly for invasive sea lamprey which are amply represented and a destructive invader of the Laurentian Great Lakes.

## Background & Summary

Dual-frequency Identification Sonar (DIDSON) is an underwater acoustic camera that has become increasingly popular in fisheries science for monitoring fish abundance and behavior in rivers and lakes^1^. For example, DIDSON has been used to determine migration timing^2^, quantify fish length^3,4^ and estimate abundance of Chinook salmon (*Oncorhynchus tshawytscha*)^5,6^, sockeye salmon (*Oncorhynchus nerka*)^2,6^ and American eel (*Anguilla rostrata*)^7^. These studies have utilized DIDSON instead of, or in conjunction with, traditional fisheries assessment tools and have benefited in many different ways. This is because DIDSON is able to capture video-like imagery under a variety of environments including those with high turbidity and low light. DIDSON also allows for continuous monitoring without manipulation of the studied organism. This is in contrast to other fisheries assessment methods such as telemetry that require the capture and subsequent release of fish and as a result the behavioral inferences made about the fish’s natural behavior are limited because tagged fish were manipulated.

DIDSON generates a significant amount of data and efficient processing of these data has posed challenges when used to assess fish abundance and behavior. As a result, manual processing of the data with traditional tools is often impractical and indeed, the development of algorithms for semi-automated processing of DIDSON data has made it easier to process these large amounts of data^3,4,8,9^. Nevertheless, fully automated fish identification is very challenging because many fish have similar body shapes and sizes and are difficult to distinguish in a DIDSON image. Presented here is a DIDSON dataset along with the methods for collecting and processing DIDSON data that could be used to create automated fish identification systems from DIDSON images. The purpose of releasing these DIDSON data is to provide the community with data that can be used to create and evaluate tools. The data are presented in their rawest form (i.e., acoustic data as collected from the DIDSON device), as well as a binary format that contains images of the visualized acoustic data. Having the data in multiple formats makes them readily available for use with either existing software such as Sound Metrics or with community developed customized tools or software. This dataset could be especially useful for helping future researchers and managers characterize the migration timing and abundance of valued and invasive fishes present in the Great Lakes and throughout North America.

## Methods

### Collecting DIDSON Data

DIDSON data were collected near the mouth of the Ocqueoc River, a tributary to Lake Huron located in Presque Isle County in northern Michigan, USA. The river is approximately 55 km long and has a 412 km^2^ drainage basin with an average daily discharge of 2.8 m^3^/s. A DIDSON camera (Standard Version 300 m) was deployed in high frequency mode (1.8 MHz) from March 20 to July 2, 2013 and March 11 to July 22, 2016. During these times, a sampling window of 10 m was used to ensure relatively high image quality for accurate fish identification when manually viewed using the Sound Metrics (V5) software. In 2013 and 2016, the DIDSON was mounted within a welded aluminum frame to protect the DIDSON from river debris. Steel chain and stainless steel hose clamps were used to secure the aluminum frame. In 2013, the DIDSON was positioned at the river mouth on the right bank (referenced looking downstream) where the channel was 14 m wide and averaged 0.9 m deep at baseflow. The field of view was positioned horizontally and perpendicular to stream flow at a downward tilt of −7.8 degree with a viewing window from 0.5 m to 10.5 m. The channel morphology within the field of view was flat with a primarily sandy substrate and the water velocity ranged from 0.20 to 0.50 m^3^/s throughout the field season. In 2016, to avoid issues with rising water levels in Lake Huron, the DIDSON camera was positioned approximately 0.28 km upstream of the river mouth 0.5 m from the left bank (referenced looking downstream). The channel at this location was approximately 23 m wide and averaged 2 m deep at baseflow. The field of view was positioned horizontally and perpendicular to stream flow at a downward tilt of −9.3 degree with a viewing window from 2.5 m to 12.5 m. The channel morphology within the viewing window was flat with a primarily sandy substrate and the water velocity ranged from 0.10 to 0.30 m^3^/s throughout the field season.

### Extracting and Labeling Targets

Data identifying and extraction for a variety of fish species within the DIDSON image is an important step towards gathering training data. Such training data could be used to develop algorithms and classifiers to automate processing of DIDSON data. Target images were collected primarily on three abundant fish species known to inhabit tributaries of northern Michigan, namely invasive sea lamprey (*Petromyzon marinus*), invasive European Carp (*Cyprinus carpio*) and native white sucker (*Catostomus commersonii*). To obtain correct identification of these species in the DIDSON data, a combination of passive integrated transponder (PIT) tag systems and underwater video cameras were used. PIT technology can detect fish anytime of the day, but is limited in that it can only detect fish that were previously captured, tagged and released. PIT technology is commonly used to track sea lamprey movement and behavior in streams^10,11^. Video cameras imaged all fishes swimming in front of the camera regardless if it contained a tag, but video observation was limited to daylight hours and therefore was selective for diurnal and crepuscular fishes.

During the 2013 DIDSON deployment in the Ocqueoc River, a PIT system (Oregon RFID, Portland, Oregon) was installed within the DIDSON’s field of view. Specifically, two PIT antennas were deployed in the Ocqueoc field of view; the first 0 m to 7 m from the DIDSON and the second 7 m to 14 m from the DIDSON. Deploying two antennas allowed us to determine approximately where the tagged sea lamprey were located in the DIDSON image. PIT-tagged sea lamprey were released in the river plume, approximately 30 m downstream of the deployment site by placing them in a cage and then opening the cage door after 4 h of acclimation. 50 males and 50 females were released during May 29, May 31, June 5, June 6, June 10, and June 11. When tagged fish swam through the DIDSON view, they were detected by the PIT antenna. Because the PIT system and the DIDSON were time synchronized, these detections were then cross-referenced with the DIDSON footage using the Sound Metrics (V5) software to observe known targets. Once known targets were identified within the DIDSON image, a short clip of 5 to 20 s containing the known target was created. To supplement the number of examples of sea lamprey, additional DIDSON data was viewed using the Sound Metrics software and additional clips were generated based on expert analysis (see Technical Validation section for full details).

**Figure 1:**
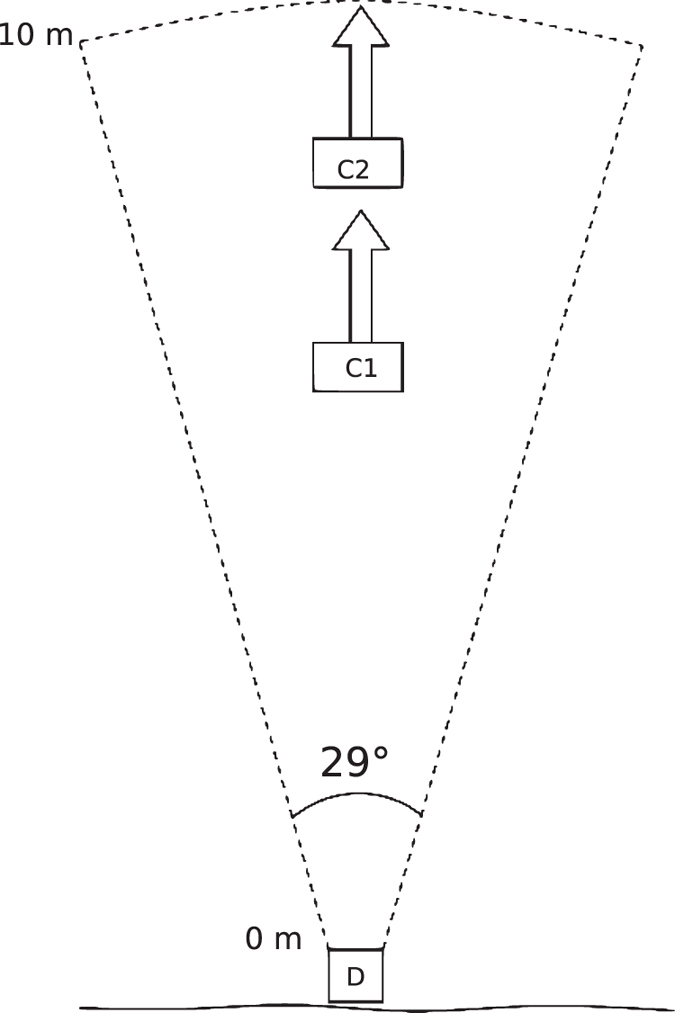
Placement of the DIDSON and video cameras for the 2016 Ocqueoc River DIDSON deployment. The placement of the cameras is denoted by C1 and C2, and they were placed 6 m and 9 m, respectively from the DIDSON, denoted as D.

**Figure 2:**
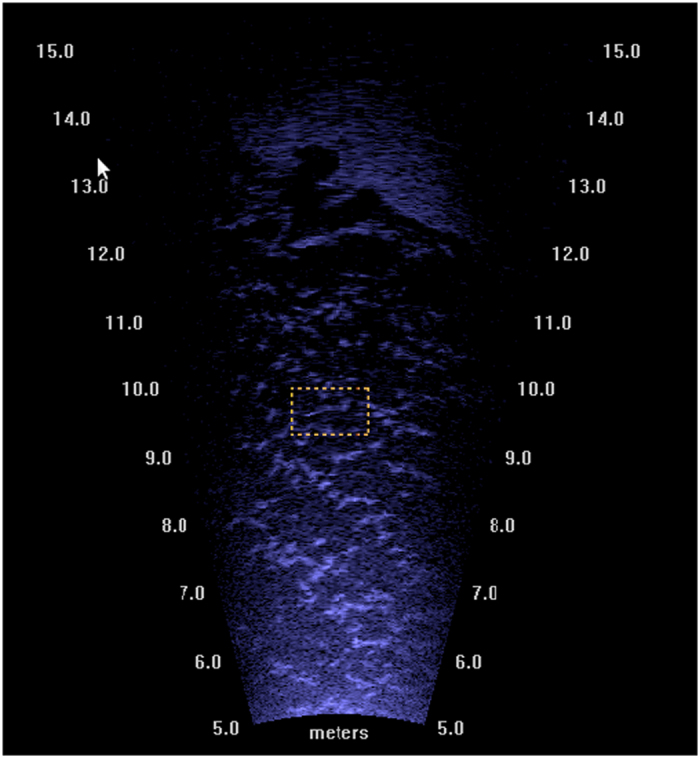
Image extracted from DIDSON data showing a sea lamprey at approximately 9.5 m from the camera. An acoustic shadow can be seen behind the fish at around 13 m. A small bounding box has been added to indicate the position of the fish.

The 2016 Ocqueoc River DIDSON deployment site was deeper and wider than the 2013 deployment site, prohibiting the use of a PIT system to obtain known targets. Therefore, during this field season, a different technique for obtaining known fish targets was used. Two video cameras were mounted approximately 1 m above the river bottom within the DIDSON field of view to ensure complete overlap of the video camera views with the DIDSON view. The cameras were positioned approximately 3 m apart (at 6 m and 9 m from the DIDSON) to minimize their overlap, which maximized the combined video camera viewing area. Both video cameras faced the same direction as the DIDSON and collected continuous video data for 2 months. Figure 1 shows the placement of the DIDSON and video cameras. These video data were manually inspected using VLC video monitoring software. Known targets identified within the video data were then cross referenced with the DIDSON data (video and DIDSON were time synchronized). Once known targets were identified within the DIDSON image, a short clip of 5 to 20 seconds containing the known target was created. Figure 2 is a sample of the visualized DIDSON acoustic data.

### Converting DIDSON Acoustic Data to Video

The DIDSON is an acoustic recording device and as a result the raw data cannot be viewed directly. The data are in binary format that starts with 512 bytes of metadata and followed by the values received from an acoustic array. These data are segmented by frames (i.e. a set of readings of the acoustic array at a particular time) and each frame starts with a header that contains information such as frame number, a time stamp, transmission mode, etc. Details on the DIDSON data file structure (i.e., how to interpret the binary data produced by a DIDSON device) was obtained by contacting the Sound Metrics corporation. The acoustic data represent reflectance of acoustic waves at a particular headings and time and can be visualized by transforming it to Cartesian coordinates and pixel intensities^12^. This conversion process can be completed using proprietary software such as Sound Metrics but such software does limit how the underlying data can be accessed. These limitations are generally two-fold. First, existing software is typically limited in terms of extensibility. This is to say that it can be difficult to incorporate new processing algorithms (e.g., different filters, automated object trackers, classifiers). Second, these existing tools were not designed for parallel processing or batch processing. Analysis of large amounts of data necessitate tools that can scale and as a result parallel processing.

To convert the acoustic data to visual data, software such as Sound Metrics can be used to export the data as a video. Alternatively, the acoustic data can also be converted to visual data (i.e., a time series of grayscale 2D images) making use of available Matlab code (see https://github.com/nilsolav/ARISreader). This software was released by Handegrad and Williams^13^ and capable of converting the acoustic data into Cartesian coordinates^12^. This existing code can be adapted to save the image data in a raw, binary form by representing it as a three-dimensional array with the third dimension being time. To facilitate the development of custom tools amenable to distributed storage and processing, the binary data was stored in a SequenceFile (i.e., an object in Hadoop for storing key-value pairs). With the image data on hand, standard image and video processing procedures can readily be applied. A Java program is provided to view the SequenceFiles and can be modified to store the data in other formats.

### Code Availability

Danner, T., Li, L., & Eickholt, J., Source code for viewing SequenceFiles, available at Open Science Framework (Data Citation 1).

Source code is available on the Open Science Framework to view the SequenceFiles created from the raw DIDSON data. The code is packaged with instructions for use. The intent of the code is to illustrate how the visualized DIDSON data is stored in the SequenceFiles and how it can be accessed. As a utility the code is licensed freely for individual, academic or commercial research. It may not be repacked or sold without written permission. Full licensing details are included with the source code.

## Data Records

The data are being provided in two formats that are the raw, acoustic data and the binary visualizations. The rationale for the multiple formats is that in some use cases it may be preferable to work with existing software (e.g., Sound Metrics) that is capable of viewing DIDSON data. In this case the acoustic data are needed. In other use cases, it may be desirable to work with data using visual processing tools and this would require image data (i.e., the visualized acoustic data). Having the data readily available in a binary format ensures that the data can be accessed without the need to convert it from its acoustic form.

The raw DIDSON data are available at Figshare via the following link under the collection name DIDSONRawFishDatasets.zip (Data Citation 2). Binary visualization data are available at the Open Science Framework under the names SEQVisualizedFishDatsets-PartI.zip, SEQVisualizedFishDatsets-PartII.zip, SEQVisualizedFishDatsets-PartIII.zip and SEQVisualizedFishDatsets-PartIV.zip (Data Citation 3). In sum, the dataset includes 3 subsets and supporting files.

### Raw DIDSON Dataset

The raw DIDSON dataset contains the original data collected by the DIDSON device on the Ocqueoc River (denoted as the “raw_DIDSON” directory). This directory contains two subdirectories that separate the data by year (i.e., OC13 and OC16 for collection in 2013 and 2016, respectively). The naming of the raw DIDSON data follows the pattern of yyyy-mm-dd_hhmmss_HF.ddf that encodes the year, month, day and the start time of collection. Each file is 30 min in duration. The extension on these files is “ddf” and these may be viewed by Sound Metrics software or via existing, community licensed software (e.g., https://github.com/nilsolav/ARISreader). Note that not all of the data collected through the DIDSON deployments are contained here. The raw data presented is limited to the files that contained identifiable targets and from which subsequent clips were generated. In total, the raw DIDSON dataset contains 105 raw DIDSON files from 2013 and 95 from 2016. These data represent approximately 100 h of data collection (i.e., around 4 days of continuous collection) and much less than was collected over the multiple month deployments. From this 100 hours of DIDSON data, 524 clips with known targets were extracted.

This folder also contains spreadsheets that describe the location of known fish in the raw DIDSON data. In 2013, the focus was on sea lamprey and in 2016 other species were also identified. As a result, all targets listed in the spreadsheets for 2013 correspond to sea lamprey. The spreadsheet for 2016 states the species of the target. All spreadsheets link the name of the longer source file (i.e, raw 30 min data file) to a smaller clip and provide details about the temporal and spatial location of the target. Spreadsheet entries for sea lamprey targets that were identified by expert analysis of the DIDSON data also include a certainty rating. This value was chosen by the expert to express confidence in the target’s identification with 3 indicating the highest level of the expert’s confidence in the identification of a sea lamprey and 1 indicating the lowest level of confidence.

### Raw DIDSON Clips

The raw DIDSON clips dataset (denoted as “raw_DIDSON_clips”) contains smaller clips of raw acoustic data that contain identified fish. These fish were identified by PIT tags, video surveillance or an expert and the clips are separated by both year of collection and identification method (i.e., the subdirectories are named OC13-by-Expert, OC13-by-PIT and OC16-by-VIDEO). Each subdirectory is further broken down by the species of fish that the clips contain. The naming of the raw DIDSON clips follows the template of yyyy-mm-dd_hhmmss_HF-S###.ddf that again encodes the year, month, day and start time of the source file. The ### is an added clip identifier that is unique for source file and can be used with the accompanying spreadsheets to reference the location of known fish in the clip. These clips were generated using the Sound Metrics software and as raw DIDSON data can be viewed using the aforementioned tools. Table 1 summarizes the number DIDSON clips with known fish by species.

### SequenceFile Dataset

The SequenceFile dataset contains a binary representation of the visualized raw acoustic data. As the raw DIDSON data represent sets of readings of the acoustic array at a particular time, it must be converted to pixel intensities on a Cartesian plane to form an image and several images in a series form a video. One means of representing a video is with a three dimensional array of unsigned bytes. Two of the dimensions represent an image and the third dimension represents the frame. Each element in the array represents a pixel’s intensity. The images here are grayscale and do not contain color information.

**Figure 3:**
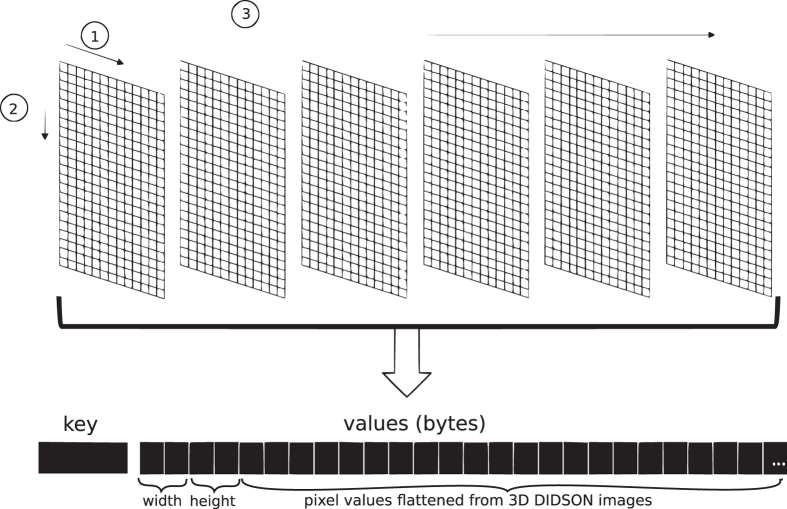
Contents of a SequenceFile for a DIDSON clip.

The container chosen to hold the converted video data was a SequenceFile. A SequenceFile is a data structure that is part of the Hadoop application programming interface (API) for storing binary files containing a series of key-value pairs. For the converted DIDSON data, the key is a String (i.e., text) that contains the source filename and the range of corresponding frames and the value is a 1D array of bytes (i.e., a flattened representation of the 3D array prepended with a 4 byte header containing the width and height of each frame). Each raw DIDSON file was segmented into sets of up to 200 frames and each set became a key-value pair (i.e., a record) in a SequenceFile. The frames in records overlap by 15 frames. This overlap allows video processing algroithms that operate over several frames to work in a distributed setting. In some applications the overlapping frames of each segment may need to be removed (i.e., ignore the first 15 frames). Figure 3 illustrates the ordering as to how the raw DIDSON data was converted to a SequenceFile. The pixels in the first frame were encoded row by row before moving to the subsequent frames.

The SequenceFile dataset was split up into 4 parts: SEQVisualizedFishDatsets-PartI.zip, SEQVisualizedFishDatsets-PartII.zip, SEQVisualizedFishDatsets-PartIII.zip and SEQisualizedFishDatsets-PartIV.zip (Data Citation 3). PartI contains the visualized data from 2013 and PartsII-IV contain the visualized data from 2016. Collectively, the contents of these 4 archives is the SequenceFile directory. As it was directly converted from the raw DIDSON clips dataset, it follows the same hierarchy. A SequenceFile can be displayed using the source code for viewing SequenceFiles. The display of SequenceFile and the original ddf file should yield the same video display. Note that while the SequenceFile format is part of the Hadoop API, a Hadoop cluster is not required to access the data. The viewer program referenced above can easily be modified to convert the data to other formats.

### Supporting Files

This folder contains two videos in AVI format of raw DIDSON files that have been converted to video. These can be played by any AVI compatible player. The videos are around 20 minutes in length, contain a number of fish and illustrate the quality of the raw data when visualized.

## Technical Validation

To collect known fish targets within the DIDSON field of view, DIDSON data were cross-referenced with PIT system data in 2013 and video camera data in 2016. A limitation of using video cameras to identify fish rather than a PIT system is that fish were only visible during daylight hours, even when infrared lights on the video cameras were activated at night. While use of video cameras provided a great opportunity to collect many known targets of white suckers and carp, they did not allow for the collection of very many sea lamprey targets because sea lamprey are nocturnal, and therefore were rarely observed during the day.

To supplement the known sea lamprey targets gathered using PIT detections (n = 65) and video data (n = 3), a trained expert manually reviewed a subset of the DIDSON data using the Sound Metrics (V5) software. Prior to manual processing, known sea lamprey targets that were gathered using the PIT system validation technique were used to train the expert reviewer, as well as test the reviewer’s accuracy at identifying sea lamprey during manual inspection of the DIDSON data. The blind reviewer watched 50 clips of DIDSON footage containing a combination of known sea lamprey images, non-target fish images, and blank images that contained only background noise. These clips were randomly selected from all days of the 2013 DIDSON deployment as well as from any time of day throughout a 24-hour period. This allowed us to evaluate whether the expert reviewer’s ability to detect sea lamprey changed over time. Of the 20 clips that contained known sea lamprey images, the expert reviewer correctly identified 19 (95% accuracy) of them, and none of the remaining 30 clips were falsely identified as containing sea lamprey.

## Usage Notes

In general the DIDSON data can be used to develop or evaluate tools that characterize the abundance or behavior of fish. Large amounts of data from extended deployments are provided that can be used to develop unsupervised machine learning tasks such as signal filtering and object tracking and the data also contains label information with the position of known targets (e.g., sea lamprey). This label information can be used to develop supervised machine learning tools such as species-specific classifiers. By providing the data in multiple formats (i.e., raw acoustic data or binary visualizations contained in SequenceFiles), it is possible to work with the data through existing software in its raw format or immediately work with the visualized data using image and video processing tools. Existing software is ill suited to handle large amounts of DIDSON data or programmatically support custom, user analyses. The purpose of releasing these DIDSON data is to provide the community with data that can be used to create and evaluate tools that can handle large amounts of data or perform custom analyses. Classifiers could be used on the existing dataset or future DIDSON datasets to better characterize abundance and behavior of many fish species in the Great Lakes or elsewhere.

Standard DIDSON software suites such as Sound Metrics or community licensed software such as the ARISreader can be used to view and access the raw DIDSON data. The visualized data created from the raw DIDSON clips can be viewed and accessed using the SequenceViewer program (Data Citation 1). This program is written in Java and can readily be modified to export the underlying image data to other formats. The SequenceFiles can also be directly accessed through the Hadoop API. To develop training and evaluation data for classifiers, the included spreadsheets can be used to pinpoint the location of specific species of fish.

The amount of labeled DIDSON data presented here may not be of sufficient size to develop machine learning tools without the aid of newer techniques to leverage commonalities among image classification tasks. Transfer learning reuses feature extractors developed on much larger labeled image sets and then repurposes them through a refining process^14^. This effectively allows large, accurate classifiers to be developed even when only a small amount of labeled images are available^15^. Additionally, through random perturbations of images (e.g., shifts, rotations, amplifications), it is possible to create additional presentations of labeled data that can be used for model construction^16^.

The SequenceViewer program (Data Citation 1) is a Java program that illustrates how the records in a SequenceFile (i.e., the individual images in a video stream) can be accessed. Each image is stored as a series of bytes that represent the intensity of a grayscale image and the SequenceViewer program simply uses this data to display an image on the screen. Instead of displaying the image, the data could be saved in a different binary format, encoded into video or used as the input to a classifier or other tool.

## Additional information

**How to cite this article**: McCann, E. *et al*. An underwater observation dataset for fish classification and fishery assessment. *Sci. Data*. 5:180190 doi: 10.1038/sdata.2018.190 (2018).

**Publisher’s note**: Springer Nature remains neutral with regard to jurisdictional claims in published maps and institutional affiliations.

## Supplementary Material



## Figures and Tables

**Table 1:** The number of raw DIDSON clips and SequenceFiles by fish species contained in the datasets.

Species	DIDSON clips and SequenceFiles
European Carp (Cyprinus carpio)	52
Largemouth bass (Micropterus salmoides)	1
Sea lamprey (Petromyzon marinus)	190
smallmouth bass	65
Steelhead (Oncorhynchus mykiss)	6
White sucker (Catostomus commersonii)	100
Brown trout (Salmo trutta)	1
Walleye (Sander vitreus)	109
